# Donor Variability and Seeding Density Shape NK-Cell Proliferation and Surface Receptor Expression: Insights from an Integrated Phenotypic and Genetic Analysis

**DOI:** 10.3390/cells14161252

**Published:** 2025-08-14

**Authors:** Neele Kusch, Jonathan Storm, Antonia Macioszek, Cornelius Knabbe, Barbara Kaltschmidt, Christian Kaltschmidt

**Affiliations:** 1 Department of Cell Biology, Bielefeld University, 33615 Bielefeld, Germany; jonathan.storm@uni-bielefeld.de (J.S.); antonia.macioszek@gmx.de (A.M.); barbara.kaltschmidt@uni-bielefeld.de (B.K.); c.kaltschmidt@uni-bielefeld.de (C.K.); 2 Forschungsverbund BioMedizin Bielefeld/OWL FBMB e.V., 33617 Bielefeld, Germany; cknabbe@hdz-nrw.de; 3 Department of Neurology, University Medical Center Göttingen, 37075 Göttingen, Germany; 4 Institute for Laboratory and Transfusion Medicine, Heart and Diabetes Centre NRW, Ruhr-University Bochum, 32545 Bad Oeynhausen, Germany; 5 Medical Faculty Ostwestfalen-Lippe, University of Bielefeld, 33615 Bielefeld, Germany; 6 Department of Molecular Neurobiology, Bielefeld University, 33615 Bielefeld, Germany

**Keywords:** natural killer cells, G-Rex culture system, seeding density, receptor expression, donor variability, flow cytometry, single-nucleotide polymorphism (SNP)

## Abstract

Natural killer (NK) cells are promising candidates for adoptive immunotherapy, but their clinical application requires standardized expansion protocols that yield functional cells in sufficient numbers. This study examined how initial seeding density and donor-intrinsic variability affect NK cell proliferation and receptor phenotype during in vitro expansion in a G-Rex^®^ 24-well plate under IL-2 stimulation. NK cells from healthy donors were analyzed longitudinally by flow cytometry, and targeted SNP sequencing of selected receptor genes (*IL2RA*, *IL2RB*, *FCGR3A*, *NCR1*, *KLRK1*, and *ICAM*-1) was performed to assess potential genetic contributions. A seeding density of 2.0 × 10^6^ cells/cm^2^ promoted high expansion rates and favorable expression of activating receptors including CD16a, NKp46, and NKG2D. Nonetheless, marked inter-donor differences were observed. Some donors exhibited impaired proliferation and aberrant receptor expression, possibly associated with high-priority SNPs and distinct haplotype structures. Others showed robust proliferation despite the absence of identifiable genetic drivers, suggesting the involvement of variants in other genes or non-genetic mechanisms such as epigenetic priming or adaptive NK-cell differentiation. These results highlight the influence of both culture conditions and donor-intrinsic factors on NK-cell expansion outcomes. Integrating phenotypic and genetic analyses may improve the reproducibility and personalization of NK-cell-based manufacturing protocols for therapeutic use.

## 1. Introduction

Natural killer (NK) cells are a key component of the innate immune system, providing rapid defense against infected or transformed cells. These lymphocytes can recognize and eliminate targets without prior sensitization, making them highly attractive for cancer immunotherapy [1,2]. NK-cell-based immunotherapies have been shown to be a promising therapeutic option in clinical trials for hematological malignancies such as acute myeloid leukemia and multiple myeloma, as well as solid tumors like renal cell carcinoma and head and neck squamous cell carcinoma [3]. For instance, a phase I trial recently demonstrated a 58.3% complete remission rate in refractory acute myeloid leukemia patients treated with ex vivo expanded NK cells, without adverse effects [4].

Despite these advances, scalable in vitro expansion and activation of NK cells remain critical challenges in fully harnessing their therapeutic potential [2]. Traditional static culture systems often face limitations in nutrient delivery, gas exchange, and scalability. The G-Rex^®^ system, featuring a gas-permeable membrane, addresses these issues by enhancing nutrient availability and gas exchange, thereby supporting efficient NK cell expansion [5]. Its scalability—from well plates to closed-system bioreactors—enables seamless transition from research optimization to clinical-grade production, including CAR-NK cells [6,7,8].

NK-cell functionality is regulated by the integrated signaling of activating and inhibitory receptors, while cytokines, most notably interleukin-2 (IL-2), promote proliferation and survival via activation of the JAK-STAT signaling pathway during in vitro expansion [9,10,11,12]. The IL-2 receptor, composed of IL-2Rα (CD25), IL-2Rβ (CD122), and the common γ-chain (CD132), exhibits variable ligand affinity and signaling potential depending on subunit composition [11,12]. Activating receptors, such as CD16a (FcγRIIIa), NKG2D, and the natural cytotoxicity receptors (NCRs), play central roles in target recognition and cytotoxic responses. Among the NCRs, NKp46 is a key receptor that recognizes viral antigens and tumor ligands [13]. CD16a mediates antibody-dependent cellular cytotoxicity (ADCC) through its interaction with the Fc region of IgG antibodies [14], while NKG2D detects stress-induced ligands such as MICA and MICB [15,16]. Additionally, ICAM-1 facilitates adhesion and immunological synapse formation, enhancing granule-mediated cytotoxicity [17,18]. The interplay of these receptors ensures effective elimination of aberrant cells while maintaining immune homeostasis. Assessing receptor expression via flow cytometry is, therefore, essential for monitoring NK-cell phenotype and functionality during expansion [19,20,21].

Genetic variations, particularly single-nucleotide polymorphisms (SNPs) within genes encoding activating NK-cell receptors, have been associated with altered receptor expression and functional capacity, potentially impacting immune surveillance and therapeutic outcomes [22,23,24,25]. For instance, the rs1049174 polymorphism in the *KLRK1* gene, which encodes NKG2D, has been linked to reduced receptor expression and impaired cytotoxicity, thereby increasing susceptibility to HPV-related cancers and influencing outcomes in hematological malignancies [22,23,24]. Additionally, variants in *FCGR3A* [25] (encoding CD16a) have been associated with modulation of ADCC responses [23,25].

To address the challenges of standardizing NK-cell expansion protocols and understanding donor variability, this study examined how initial seeding density and donor-intrinsic factors affect NK-cell proliferation and surface receptor expression in a G-Rex^®^ 24-well system under IL-2 stimulation. Additionally, we performed targeted SNP analysis of key activating receptors and IL-2 receptor subunits to explore potential genetic contributions. This integrated phenotypic and genotypic approach offers mechanistic insight into donor-specific differences and supports the refinement of NK-cell manufacturing strategies for therapeutic application.

## 2. Materials and Methods

### 2.1. Isolation and Cultivation of NK Cells

#### 2.1.1. Isolation of NK Cells from Peripheral Blood

NK cells were isolated directly from buffy coats obtained from healthy donors (Table 1). The buffy coats were provided by the Institut für Laboratoriums- und Transfusionsmedizin (HDZ NRW, Bad Oeynhausen, Germany), where blood donations were processed in accordance with the German Hemotherapy Guidelines. Whole blood was centrifuged as part of standard blood processing, and the intermediate leukocyte-rich layer (buffy coat) was separated using a blood-bag expressor. This fraction contains leukocytes, platelets, residual erythrocytes, and plasma. For NK-cell enrichment, the RosetteSep™ Human NK Cell Enrichment Cocktail (Stemcell Technologies Germany GmbH, Cologne, Germany) was added directly to the buffy coat. NK cells were then isolated by density-gradient centrifugation using SepMate™-50 tubes and Lymphoprep™ (both from Stemcell Technologies, Cologne, Germany), according to the protocol provided with the RosetteSep™ enrichment kit.

#### 2.1.2. Seeding and Cultivation in G-Rex^®^ 24-Well Plate

NK cells obtained from five healthy donors were seeded in a 24-well G-Rex^®^ plate (Wilson Wolf, Saint Paul, MN, USA) at four defined initial densities—0.5 × 10^6^, 1.0 × 10^6^, 2.0 × 10^6^, and 2.5 × 10^6^ NK cells per cm^2^, with each well providing a growth surface of 2 cm^2^. Except for the removal of cells through sampling, as described below, no adjustments to cell density were made throughout the 49-day culture period, and cells were maintained under static conditions without splitting or dilution. Remaining donor cells were cryopreserved in 10% dimethyl sulfoxide (DMSO; AppliChem, Darmstadt, Germany) and stored for downstream applications, such as DNA extraction.

Cells were cultured in 8 mL NK MACS^®^ Basal Medium supplemented with the corresponding NK MACS^®^ Supplement (both Miltenyi Biotec, Bergisch Gladbach, Germany), 1% penicillin–streptomycin (Sigma-Aldrich GmbH, Steinheim, Germany), 5% human AB serum (Pan-Biotech, Aidenbach, Germany), and IL-2 Premium Grade (Miltenyi Biotec, Bergisch Gladbach, Germany) in the amount specified below. Cultures were incubated at 37 °C in a humidified atmosphere containing 5% CO_2_.

Flow cytometric analyses were performed at multiple intervals, as indicated in the corresponding figures. Cell density and viability were monitored for the entire cultivation period, whereas receptor expression analyses were only conducted during the first 25 days. On each measurement day, the plate was placed under the workbench for 10 min to allow cell sedimentation onto the gas-permeable membrane. Subsequently, 6 mL of the supernatant was carefully removed. The remaining 2 mL of cell suspension was resuspended thoroughly, and 200 µL (corresponding to 10% of the total volume) was taken for flow-cytometric staining. After sample collection, the wells were replenished with 6.2 mL of fresh medium to a final volume of 8 mL. A total of 4000 U of IL-2 was added to achieve a final concentration of 500 U/mL fresh IL-2. Accordingly, the medium and IL-2 were changed every 3 to 4 days.

### 2.2. Flow Cytometric Analysis Using an 8-Color Staining Panel

Flow cytometry was used to evaluate the impact of initial seeding densities and inter-donor variability on the NK-cell phenotype during cultivation. An 8-color antibody panel was employed to characterize surface markers associated with NK-cell identity, activation, and adhesion (Table 2). In this context, expression levels were analyzed with respect to both donor origin and initial seeding density. Data acquisition was performed using a Gallios™ Cell Analyzer—10 Colors, 3 Lasers (Beckman Coulter Life Sciences, Krefeld, Germany).

#### 2.2.1. Sample Preparation and Measurement

For quantification of viable lymphocytes and assessment of surface receptor expression, two distinct staining strategies were used. Throughout the entire 49-day cultivation period, 4’,6-diamidin-2-phenylindol (DAPI) was applied for viability staining, and CountBright™ Absolute Counting Beads (Thermo Fisher Scientific Life Technologies, Darmstadt, Germany) were added for cell quantification. In addition, during the first 25 days of culture, cells from each condition were stained with fluorochrome-conjugated antibodies in the presence of a tandem signal enhancer (Stemcell Technologies, Cologne, Germany) to assess surface marker expression. Excess cell suspension not used for flow cytometric staining was pelleted and stored at −80 °C for future analyses.

#### 2.2.2. Gating Strategy

To ensure accurate analysis, gating was initiated by the selection of stable flow regions to exclude technical artifacts and ensure consistent event acquisition. CountBright™ Absolute Counting Beads were used to determine the acquisition volume and enable subsequent cell quantification. Beads were excluded, and further analysis was performed on the remaining cell population. Debris and doublets were removed through a series of gating steps. Singlets were then gated, and lymphocytes were identified based on their characteristic forward and side-scatter properties. Viable lymphocytes were subsequently selected by DAPI exclusion. An illustration of the gating strategy is available in a previously published study [10].

#### 2.2.3. Calculation of Viable Lymphocyte Numbers

The acquisition volume of each sample was determined using CountBright™ Absolute Counting Beads. Based on this volume and the number of events corresponding to viable lymphocytes, the total cell count per well was calculated. To account for the 10% of cell suspension removed at each measurement time point, this loss was mathematically adjusted. The resulting values represent the estimated total number of viable lymphocytes per well, adjusted to account for prior sample removal.

#### 2.2.4. Determination of NK Cell Purity

To determine NK cell purity, CD45^+^ cells were gated within the DAPI^−^ lymphocyte population. CD3^+^ cells were then excluded, and CD56^+^ cells were identified within the CD3^−^ fraction. Purity was calculated by multiplying the proportion of lymphocytes within the DAPI^-^ fraction with the proportion of CD3^−^ cells and, subsequently, with the percentage of CD56^+^ cells among the CD3^−^ population. This approach yields the relative frequency of CD3^−^CD56^+^ NK cells within the overall viable cells in the sample in percent.

#### 2.2.5. Analysis of NK-Cell Receptor Expression

NK-cell receptor expression was assessed by determining both the percentage of receptor-positive cells and the corresponding mean fluorescence intensity (MFI) within the CD3^−^CD56^+^ NK-cell population.

### 2.3. Genetic Analysis for SNP Identification

#### 2.3.1. DNA Extraction

Genomic DNA was isolated using the NucleoBond™ HMW DNA kit (Macherey-Nagel, Düren, Germany), which utilizes anion exchange chromatography for the purification of high-molecular-weight DNA. All steps were carried out according to the manufacturer’s instructions. DNA concentration and purity were assessed by UV/Vis spectrophotometry and fluorometric quantification. DNA integrity was evaluated by agarose gel electrophoresis.

#### 2.3.2. DNA Sequencing, Basecalling, Mapping, SNP Calling, and Annotation

For each sample, gDNA was sheared to a length of ~12 kb using a gTube (Covaris Inc., Woburn, MA, USA) according to the manufacturer’s instructions. Shorter fragments were removed using the SRE Short Read Eliminator Kit (PacBio, Menlo Park, CA, USA) according to the manufacturer’s instructions. For each sample, a barcoded library was prepared from the sheared and purified gDNA using the SQK-NBD114.24 kit (Oxford Nanopore Technologies (ONT), Oxford, UK). The pooled libraries were sequenced on a PromethION sequencer using a FLO-PRO114M flowcell (ONT) using adaptive sampling in enrichment mode. The regions to be enriched were the genes under study (*IL2RA*, *IL2RB*, *FCGR3A*, *NCR1*, *KLRK1*, and *ICAM1*), including 25 kb up- and downstream. Basecalling was performed with dorado v7.6.7 and basecalling model dna_r10.4.1_e8.2_400bps_sup_v4.3.0. The basecalled reads were trimmed on both ends with cutadapt v4.8 [26] with parameters “-e 0.2 -O 15 --trimmed-only -g AAGGTTAANNNNNNNNNNNNNNNNNNNNNNNNCAGCACCT” for the 5’-end and “-O 15 -e 0.2 -a AGGTGCTGNNNNNNNNNNNNNNNNNNNNNNNNTTAACCTTAGCAAT -m 1000” for the 3’-end. The trimmed reads were mapped to the GRCh38 reference genome using minimap2 v2.27-r1193 [27], with parameters “-a -x lr:hq --secondary=no --sam-hit-only” and sorted using SAMtools sort v1.19.2 [28]. SNPs were called using Clair3 v1.10.0 [29], with parameters “--platform=“ont” --model_path=r1041_e82_400bps_sup_v430 --bed_fn=GOI.bed --qual=2 --var_pct_full=0.7 --ref_pct_full=0.1 --snp_min_af=0.08 --indel_min_af=0.15 --print_ref_calls --gvcf”. The individual gVCF files were merged with BCFtools merge v1.19.1 [30], with parameters -0 -O v -g hg38.fa.gz. Finally, the combined VCF file was annotated using SnpEff v5.2e [31] with database GRCh38.99 and SnpSift Annotate v5.2e [32] with the ClinVar database [33].

### 2.4. Analysis and Illustration

#### 2.4.1. Software

Flow cytometric measurements were performed using Kaluza for Gallios™ software (version 1.2), and NK-cell identification was carried out with Kaluza for Analysis (version 1.2) (both from Beckman Coulter Life Sciences, Krefeld, Germany), as described in Section 2.2.4. Receptor expression data were analyzed using CellEngine^®^ (CellCarta, Montreal, QC, Canada), as outlined in Section 2.2.5. Cell proliferation analysis was conducted in Microsoft Excel (version 2211; Microsoft, Redmond, WA, USA), while data visualization was performed using GraphPad Prism (version 8.3.0, GraphPad Software, Boston, MA, USA).

#### 2.4.2. Statistical Analyses

To assess the effects of initial seeding density, donor identity, and culture duration on total lymphocyte count and NK-cell fold expansion, statistical analyses were performed using two-way analysis of variance (ANOVA). Depending on the experimental design, either seeding density or donor was used as the main column factor, while culture day served as the row factor in all models. For post hoc comparisons of group means, Tukey’s multiple comparisons test was applied with a significance threshold of *p* < 0.05. Statistical significance was indicated using the following standard notation: *ns* (not significant), *p* < 0.05 (*), *p* < 0.01 (**), *p* < 0.001 (***), and *p* < 0.0001 (****). Only statistically significant comparisons are shown; non-significant comparisons were omitted for clarity. All statistical analyses were performed using GraphPad Prism (version 8.3.0, GraphPad Software, Boston, MA, USA). Statistical results are presented as mini-tables embedded in the figure panels, summarizing relevant *p*-values and comparisons.

## 3. Results

Primary NK cells from peripheral blood of five healthy donors were cultured in a G-Rex^®^ 24-well plate for approximately 50 days. Each donor was seeded at the following four distinct initial densities: 0.5 × 10^6^, 1.0 × 10^6^, 2.0 × 10^6^, and 2.5 × 10^6^ NK cells per cm^2^. The densities were not readjusted throughout the cultivation period. NK-cell expansion was monitored over a 49-day period, while receptor expression and NK-cell purity were monitored during the first 25 days of culture, using an eight-color flow cytometry panel (CD45, CD3, CD56, CD16a, NKp46, NKG2D, and ICAM-1).

### 3.1. NK-Cell Proliferation Is Dependent on Seeding Density and Markedly Influenced by Donor-Intrinsic Factors

**Figure 1:**
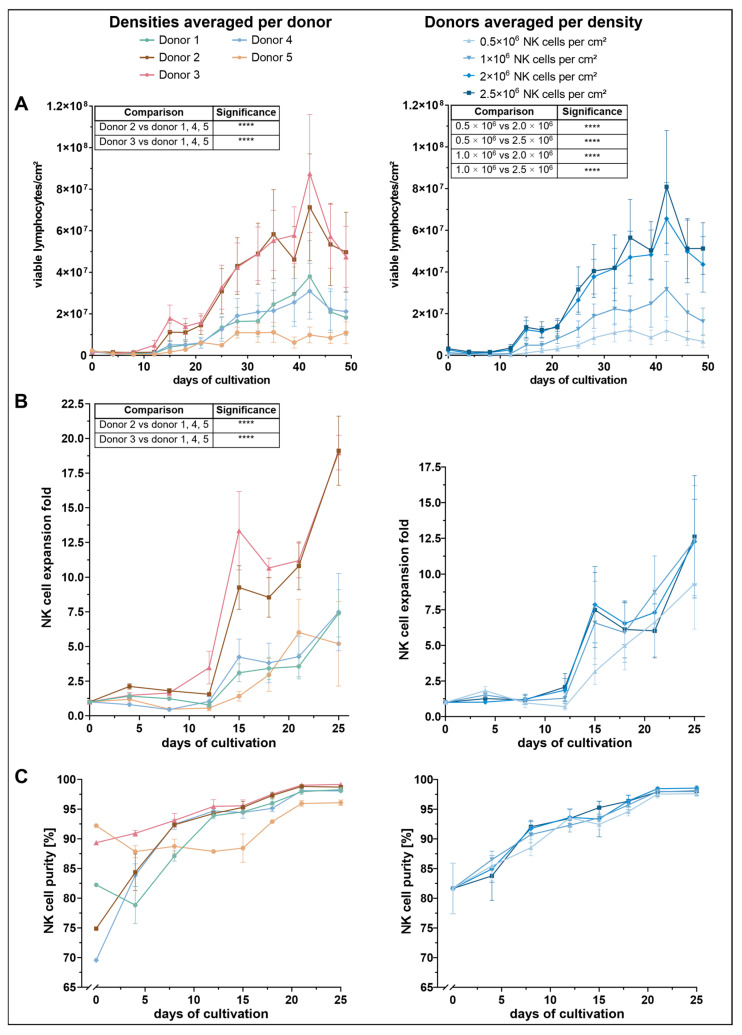
NK cells were isolated from five individual donors and seeded at four different initial densities in a 24-well G-Rex^®^ plate cultured for 49 days: (**A**) number of viable lymphocytes per cm^2^, grouped by donor (**left**) and by density (**right**); (**B**) NK-cell expansion relative to day 0, grouped by donor (**left**) and by density (**right**); (**C**) NK-cell purity (% CD56^+^CD3^−^ among CD45^+^ cells), grouped by donor (**left**) and by density (**right**). All data are presented as the mean ± SEM. The results of statistical comparisons for lymphocyte counts and NK-cell expansion folds (Tukey’s post hoc test after two-way ANOVA, as detailed in Section 2.4.2) are shown as mini-tables embedded within each panel. Only statistically significant comparisons (*p* < 0.05) are shown; non-significant comparisons were omitted for clarity (*p* < 0.0001 (****)).

To assess the impact of initial seeding density on NK-cell proliferation, viable lymphocyte counts were monitored throughout the culture period (Figure 1A). Across all conditions, a similar proliferation pattern was observed: cell numbers initially declined until approximately day 8, followed by a steady increase until day 32, after which cell numbers began to fluctuate. Among the tested conditions, cultures seeded with 2.0 × 10^6^ and 2.5 × 10^6^ NK cells per cm^2^ demonstrated the most robust expansion, reaching peak lymphocyte densities of approximately 7–8 × 10^7^ cells per cm^2^ by day 42. These two conditions yielded significantly higher cell counts than the lower seeding densities of 0.5 × 10^6^ and 1.0 × 10^6^, while no significant difference was observed between 2.0 × 10^6^ and 2.5 × 10^6^.

Proliferation was further influenced by donor-specific variation. Donors 2 and 3 exhibited a markedly higher expansion profile than the other donors. Both showed a pronounced proliferative burst from around day 21, reaching maximum viable cell densities of 7.1 × 10^6^ (donor 2) and 8.8 × 10^6^ cells/cm^2^ (donor 3) by day 42. These two donors significantly outperformed donors 1, 4, and 5, though no significant difference was detected between donor 2 and donor 3 themselves. In contrast, donor 5 showed the lowest proliferative response, reaching a peak of only 1.1 × 10^7^ viable cells between days 28 and 35. Despite this weaker performance, statistical analysis revealed no significant differences between donor 5 and donors 1 or 4.

Consistent with total lymphocyte counts, NK-cell expansion was dependent on both initial seeding density and donor (Figure 1B). Among the four tested input concentrations, the lowest seeding density (0.5 × 10^6^ NK cells/cm^2^) resulted in the weakest expansion response, reaching a maximum mean-fold NK-cell expansion of 9.3 on day 25. The other three densities yielded higher NK-cell expansion rates, with peak values ranging between 12.3 and 12.6. Despite a visible trend toward improved expansion at higher densities, statistical analysis revealed no significant differences between conditions.

In contrast, NK-cell expansion varied significantly across donors. Donors 2 and 3 exhibited the most robust NK-cell expansion profiles. Donor 3 initially showed a steeper increase, reaching a mean fold expansion of 13.3 on day 15, compared to 9.3 in donor 2. However, by day 25, both donors reached comparable maxima of approximately 19. Statistical analysis revealed that donors 2 and 3 exhibited significantly greater NK-cell expansion compared to donors 1, 4 and 5. Notably, no significant difference was observed between the expansion of donor 2 and donor 3.

Donors 1 and 4 showed moderate NK-cell expansion levels, with maximum values of approximately 7.5 on day 25. Donor 5 showed the lowest overall NK-cell expansion but reached its peak earlier, with a maximum fold expansion of 6 on day 21. However, these differences were not statistically significant when compared to the moderately expanding donors.

Seeding density did not significantly affect NK-cell purity during the first 25 days of culture (Figure 1C). Instead, notable differences were observed between donors, particularly in the early phase immediately following NK-cell isolation. Donor 5 exhibited the highest initial NK-cell purity (92%), followed by donor 3 (89%). In contrast, donors 2 and 4 started at lower purity levels ranging between 70% and 75%, whereas donor 1 showed an intermediate value of 82%. By day 8, NK-cell purity had equalized among donors 2, 3, and 4. Donor 1 reached comparable levels by day 12. Donor 3 achieved the highest purity overall, reaching 99% on day 25. From day 8 onward, donor 5 consistently exhibited the lowest NK-cell percentages, despite its initially highest purity. Nonetheless, purity in donor 5 stabilized at ~96% by day 25, whereas all other donors exceeded ~98%.

### 3.2. Expression of Activating Receptors Varies with Seeding Density and Shows Distinct Donor-Specific Profiles

To further characterize the cultured NK cells, the expression dynamics of the activating receptors CD16a, NKp46, and NKG2D, along with the adhesion molecule ICAM-1, were analyzed over the first 25 days of the cultivation period. Both the percentage of receptor-positive cells and the corresponding mean fluorescence intensity (MFI) within the CD3^−^CD56^+^ NK-cell population were assessed.

**Figure 2:**
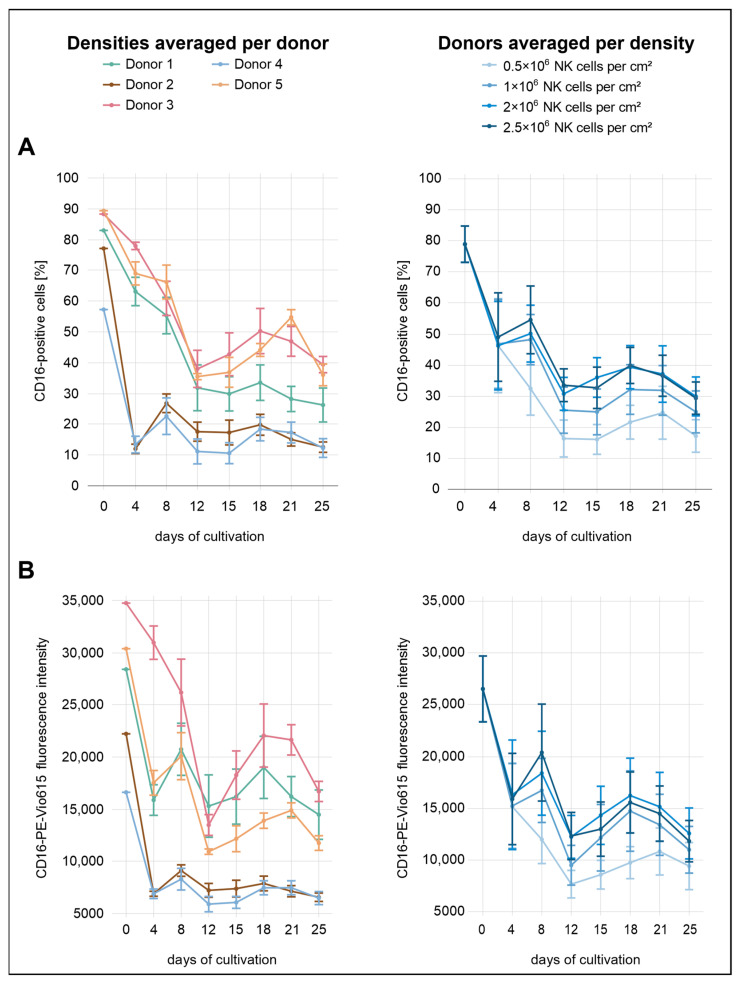
NK cells were isolated from five individual donors and were seeded at four different densities in a 24-well G-Rex^®^ plate. CD16a expression was assessed by flow cytometry over a period of 25 days: (**A**) proportion of CD16a^+^ NK cells (CD56^+^CD3^−^), grouped by donor (**left**) and by seeding density (**right**), shown as the mean ± SEM; (**B**) mean fluorescence intensity (MFI) of CD16a in NK cells (CD56^+^CD3^−^), grouped by donor (**left**) and by seeding density (**right**), shown as the mean ± SEM.

Immediately after isolation, the proportions of CD16a^+^ NK cells were high across all conditions, averaging 79% (Figure 2). By day 4, expression levels had dropped markedly, with all densities showing a decrease to approximately 46–49%. From this point onward, CD16a expression fluctuated, showing an overall downward trend. While no substantial differences were observed between the two highest seeding densities (2.0 × 10^6^ and 2.5 × 10^6^ cells/cm^2^), lower initial densities were consistently associated with reduced CD16a expression, both in terms of the percentage of positive cells and MFI.

Considerable variation in CD16a expression was observed between donors. Donors 2 and 5 exhibited the lowest initial expression levels, with 77% and 57% CD16a^+^ cells, respectively. Notably, donor 4 showed the lowest expression across all donors. By day 4, all three donors had already declined to approximately 12%, followed by a modest recovery to 23–27% by day 8. In the subsequent days, CD16a^+^ cell proportions in donors 2 and 4 remained within a relatively narrow range of 11–20% until the end of the analysis period. Among all donors, donor 3 and donor 5 initially exhibited the highest percentages of CD16a^+^ cells, reaching 88% and 89%, respectively. In both donors, a decline was observed during the first 12 days, with values dropping to 38–39%. Donor 3 displayed a transient recovery to 50% by day 18, followed by a slight decrease to 39% on day 25. Donor 5 followed a similar trajectory in the percentage of CD16a^+^ cells but differed in MFI dynamics; initial values were higher than those of donor 1 but declined more steeply after day 8. While most donors exhibited a secondary peak for both parameters around day 18, donor 5 showed a delayed peak on day 21. Donor 1 showed moderate CD16a expression throughout the analysis period, with values generally positioned below donors 3 and 5 but above donors 2 and 4. By day 25, the proportion of CD16a^+^ cells in donor 1 reached 26%. In terms of MFI, donor 1 remained below donor 3 and was slightly lower than donor 5 until day 8 but exceeded donor 5 thereafter. At the end of the observation period (day 25), donors 3 and 5 showed the highest percentages of CD16a^+^ cells (36–39%), followed by donor 1 (25%). Donors 2 and 4 exhibited the lowest final expression levels, with only 12–13% CD16a^+^ cells remaining.

**Figure 3:**
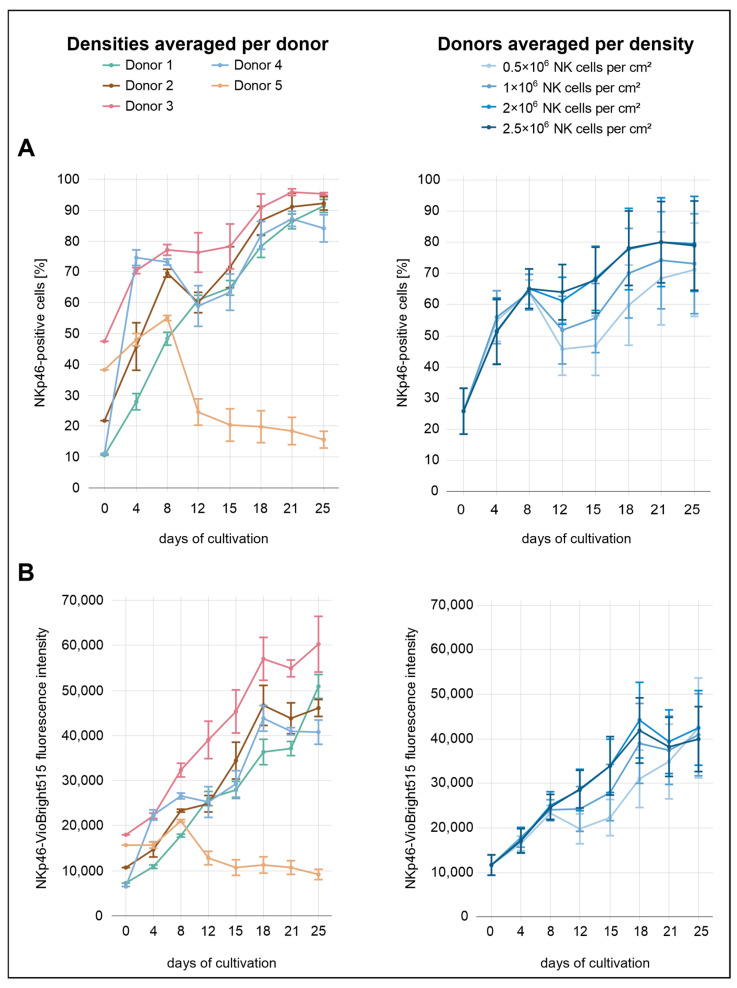
NK cells were isolated from five individual donors and were seeded at four different densities in a 24-well G-Rex^®^ plate. NKp46 expression was assessed by flow cytometry over a period of 25 days. (**A**) Proportion of NKp46^+^ NK cells (CD56^+^CD3^−^), grouped by donor (**left**) and by seeding density (**right**), shown as mean ± SEM. (**B**) Mean fluorescence intensity (MFI) of NKp46 in NK cells (CD56^+^CD3^−^), grouped by donor (**left**) and by seeding density (**right**), shown as mean ± SEM.

During the initial eight days of cultivation, NKp46 expression did not appear to be influenced by seeding density (Figure 3). From day 8 onward, however, lower seeding densities (0.5 × 10^6^ and 1.0 × 10^6^ NK cells/cm^2^) were associated with reduced NKp46 expression, whereas no clear differences were observed between the two highest densities (2.0 × 10^6^ and 2.5 × 10^6^ NK cells/cm^2^). The proportion of NKp46^+^ cells increased markedly from 26% to approximately 65% by day 8, followed by a slight decline. At the highest density (2.5 × 10^6^ cells/cm^2^), however, expression levels remained relatively stable and did not decrease. In contrast, NKp46 MFI remained largely stable across all densities, with a decrease only evident at the lowest density (0.5 × 10^6^ cells/cm^2^). From day 12 onward, both the proportion of NKp46^+^ cells and the MFI showed a renewed increase. The percentage of NKp46^+^ cells approached a plateau toward the end of the culture period, stabilizing between approximately 70% and 80%, particularly in the two highest seeding densities (2.0 × 10^6^ and 2.5 × 10^6^ NK cells/cm^2^). In contrast, the MFI showed a transient decline at day 18 in these two seeding conditions, and a similar—but less pronounced—drop was also observed at the initial seeding density of 1.0 × 10^6^ cells/cm^2^. This drop was not observed at the lowest seeding density (0.5 × 10^6^ cells/cm^2^).

A comparison across donors revealed markedly distinct expression profiles. Donor 5 initially showed a moderate proportion of NKp46^+^ cells (38%) compared to the other donors. This was followed by a sharp increase until day 8, and then a steep decline to approximately 25% by day 12. This downward trend continued gradually, reaching 16% by day 25. In contrast, donor 3 exhibited the highest proportion of NKp46^+^ cells immediately after isolation (48%) and consistently maintained elevated percentages throughout the culture period, peaking at 96% on day 21. This strong expression profile was also reflected in the MFI values. Donors 1 and 4 started with the lowest proportions of NKp46^+^ cells (11%). Donor 4 showed the steepest early increase, reaching 75% by day 4. A pronounced decline followed, with expression dropping to 59% on day 12, after which levels began to rise again. In contrast, donor 1 exhibited the most gradual and continuous increase throughout the entire cultivation period, reaching 91% NKp46^+^ cells on day 25. Donor 2 showed an intermediate expression profile, starting at approximately 22%. From day 12 onward, NKp46 expression remained consistently between the levels observed in donor 3 and those of the remaining donors.

While NKp46 expression dynamics were relatively similar between the proportion of receptor-positive cells and MFI across the different seeding densities, more pronounced differences emerged when comparing these parameters across donors. MFI values generally showed a more uniform upward trajectory with fewer fluctuations, whereas the proportion of NKp46^+^ cells varied markedly among donors.

**Figure 4:**
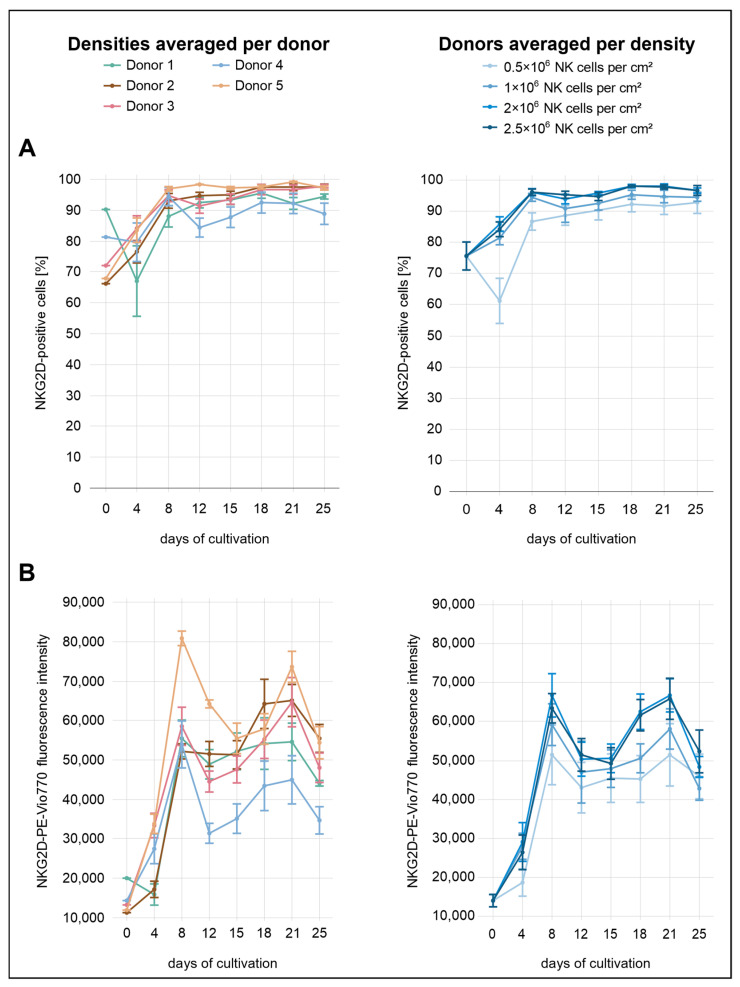
NK cells were isolated from five individual donors and were seeded at four different densities in a 24-well G-Rex^®^ plate. NKG2D expression was assessed by flow cytometry over a period of 25 days: (**A**) proportion of NKG2D^+^ NK cells (CD56^+^CD3^−^), grouped by donor (**left**) and by seeding density (**right**), shown as the mean ± SEM; (**B**) mean fluorescence intensity (MFI) of NKG2D in NK cells (CD56^+^CD3^−^), grouped by donor (**left**) and by seeding density (**right**), shown as the mean ± SEM.

Immediately after isolation, the average proportion of NKG2D^+^ NK cells across all conditions was 76% (Figure 4). Distinct differences between the seeding densities were already evident by day 4, with the exception of the two highest densities (2.0 × 10^6^ and 2.5 × 10^6^ NK cells/cm^2^). Higher initial seeding densities were associated with higher levels of NKG2D expression, both in terms of the proportion of expressing cells and the surface density of the receptor (MFI). At day 4, the lowest density condition (0.5 × 10^6^ cells/cm^2^) showed a decline to 61% NKG2D^+^ cells, whereas all other densities displayed increasing proportions. In terms of MFI, the increase at this density was less pronounced than in the other conditions. By day 8, the lowest density (0.5 × 10^6^ cells/cm^2^) had reached 87%, whereas the second-lowest (1.0 × 10^6^ cells/cm^2^) and the two highest densities (2.0 × 10^6^ and 2.5 × 10^6^ NK cells/cm^2^) showed stronger expression levels, reaching 94% and 96%, respectively. From day 8 onward, the percentage of NKG2D^+^ cells remained relatively stable. At the final measurement point on day 25, values were high across all conditions, with 93% at the lowest density (0.5 × 10^6^ cells/cm^2^), 95% at the second-lowest (1.0 × 10^6^ cells/cm^2^), and 97% at the two highest densities (2.0 × 10^6^ and 2.5 × 10^6^ NK cells/cm^2^). In contrast, MFI values followed a different pattern, rising markedly until day 8, followed by a transient decrease. From day 15 onward, MFI increased again, peaking around day 21 before declining more noticeably by day 25.

Donor-specific expression dynamics were consistent with the density-dependent trends, though with more pronounced inter-individual differences. Donor 1 displayed the highest proportion of NKG2D^+^ cells at baseline (90%), but both the proportion of expressing cells and the MFI declined notably by day 4. In contrast, donors 2 and 5 initially exhibited the lowest levels, with 66% and 68% NKG2D^+^ cells, respectively. Both donors showed a marked increase by day 4, reaching 77% (donor 2) and 84% (donor 5). The corresponding MFI values also increased, more steeply in donor 5 than in donor 2. While the proportion of NKG2D-expressing cells in donor 4 changed only slightly during the initial days (from 81% to 80%), the MFI showed a more pronounced increase.

Notably, donor 4 was the only individual to exhibit a marked decrease in the percentage of NKG2D^+^ cells on day 12 and consistently showed the lowest overall NKG2D expression among all donors, particularly in terms of surface density. By day 25, the proportion of NKG2D^+^ cells in donor 4 reached only 89%, whereas all other donors exceeded 94%. Donor 5 consistently showed the highest NKG2D expression across nearly the entire analysis period. From day 8 onward, the percentage of NKG2D^+^ cells in this donor remained above 97%. In terms of MFI, the greatest separation between donor 5 and the other donors was observed on day 8, whereas the largest difference in the proportion of NKG2D^+^ cells occurred on day 12. On that day, donor 5 reached 98% NKG2D^+^ cells. The next highest value was observed in donor 2 with 95%, followed by donor 1 with 92%, donor 3 with 91%, and donor 4, who showed the lowest value at 84%. At the same time point, MFI values decreased in all donors, with donors 4 and 5 showing more pronounced reductions.

**Figure 5:**
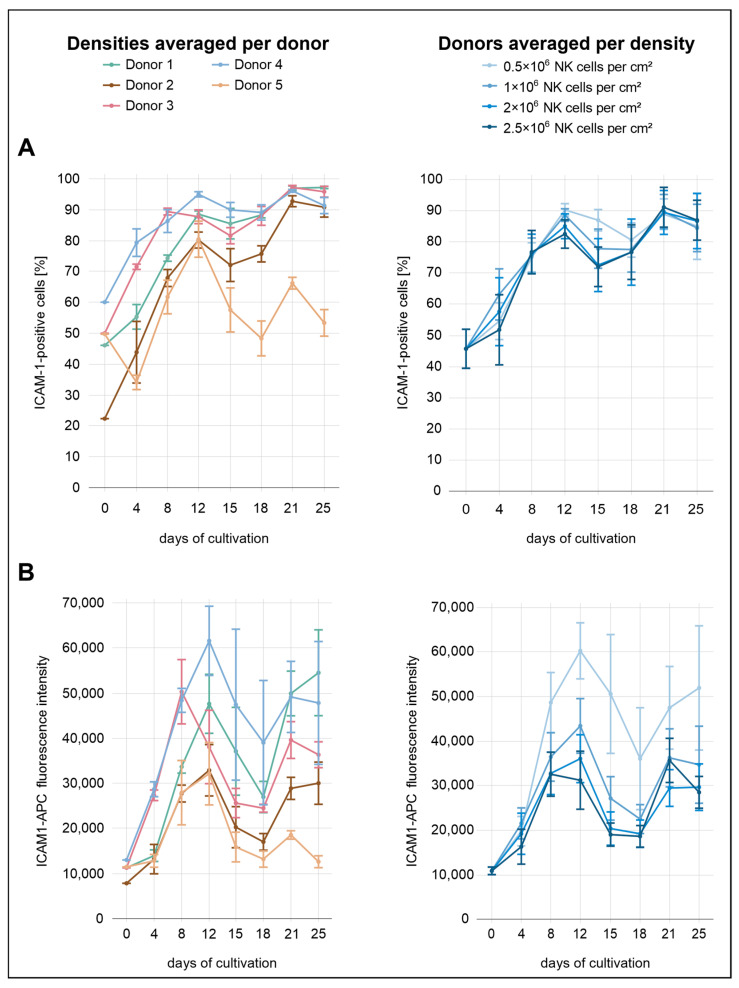
NK cells were isolated from five individual donors and were seeded at four different densities in a 24-well G-Rex^®^ plate. ICAM-1 expression was assessed by flow cytometry over a period of 25 days: (**A**) proportion of ICAM-1^+^ NK cells (CD56^+^CD3^−^), grouped by donor (**left**) and by seeding density (**right**), shown as the mean ± SEM; (**B**) mean fluorescence intensity (MFI) of ICAM-1 in NK cells (CD56^+^CD3^−^), grouped by donor (**left**) and by seeding density (**right**), shown as the mean ± SEM.

Across all conditions, the percentages of ICAM-1^+^ cells were relatively similar, with only minor differences between the seeding densities (Figure 5). From the start of cultivation until day 12, a gradual increase was observed across all seeding conditions, with ICAM-1^+^ cell proportions rising from 46% after isolation to 82% at 2.0 × 10^6^ cells/cm^2^, 85% at 2.5 × 10^6^ cells/cm^2^, 89% at 1.0 × 10^6^, and 90% at 0.5 × 10^6^ cells/cm^2^. From day 12 to day 15, ICAM-1 expression decreased slightly in the three highest seeding densities. This was followed by a renewed increase, with expression levels reaching 89–91% on day 21 before declining to 84–87% by day 25. In contrast, the decline in ICAM-1 expression at the lowest density occurred later, becoming apparent at day 18 rather than day 15. In terms of MFI, more pronounced differences between the seeding densities were observed. The lowest density consistently showed the highest MFI throughout the cultivation period, indicating that reduced cell density was associated with increased ICAM-1 expression at the per-cell level.

The donor-specific profiles were more heterogeneous. Donor 4 exhibited the highest overall ICAM-1 expression over the analysis period, both in terms of MFI and the percentage of positive cells. This donor also exhibited the highest ICAM-1 expression immediately after isolation, with 60% ICAM-1^+^ cells. Both parameters showed a local peak on day 12, with 95% ICAM-1^+^ cells and the highest surface density observed. While the percentage increased slightly to 96% by day 25, surface density declined thereafter. Donor 3 initially followed a similar expression profile to donor 4, with elevated values for both parameters until day 8. From day 12 onward, the proportion of ICAM-1^+^ cells in donor 3 closely matched that of donor 1. For example, both donors reached approximately 88% on day 12 and 97% on day 21. Donor 5 initially displayed relatively high values for both MFI and the proportion of ICAM-1^+^ cells (50%) but subsequently showed the lowest ICAM-1 expression from day 12 onward. This decline was especially evident in the proportion of ICAM-1^+^ cells, which dropped to 53% by day 25, whereas all other donors remained between 91% and 97%. Donor 2 exhibited the second-lowest ICAM-1 expression over the course of the analysis period, both in terms of MFI and the proportion of ICAM-1^+^ cells. Although the MFI remained comparatively low throughout the cultivation, the percentage of ICAM-1^+^ cells gradually increased, approaching levels observed in other donors from day 21 onward.

### 3.3. SNPs in Selected Receptor Genes Show Donor-Specific Profiles but Do Not Sufficiently Explain Observed Donor Differences

**Figure 6:**
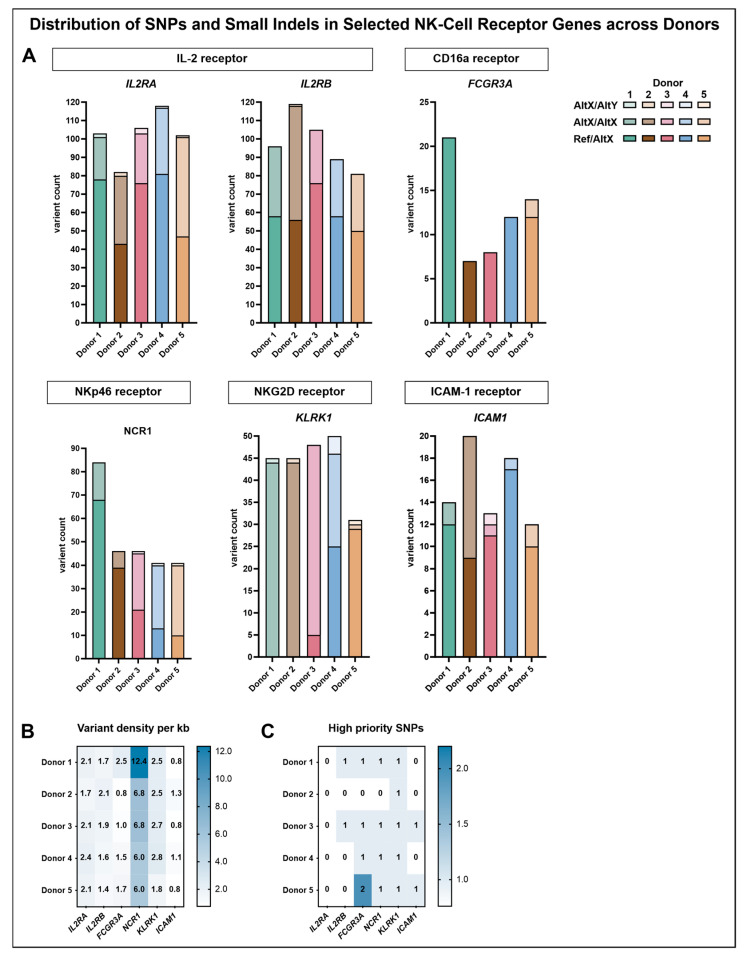
Distribution of small indels and SNPs across selected NK-cell receptor genes in sequenced donors (*n* = 5): (**A**) stacked bar plots illustrating the distribution of detected variants per receptor gene, separated into heterozygous genotypes with one reference allele (Ref/AltX), homozygous alternate genotypes (AltX/AltX), and heterozygous with two different alternate alleles (AltX/AltY); (**B**) heatmap showing the distribution of all detected variants, normalized to the number of variants per 1000 base pairs for each gene; (**C**) heatmap displaying the absolute number of high-priority SNPs, defined as variants with a predicted missense effect and moderate functional impact.

To complement the phenotypic characterization of NK-cell donors, a targeted variant analysis was performed to assess whether genetic polymorphisms within the corresponding receptor genes might contribute to the observed donor-specific differences. The analysis focused on SNPs and small insertions/deletions (indels) within the genomic regions of CD16a (*FCGR3A*), NKp46 (*NCR1*), NKG2D (*KLRK1*), and ICAM-1 (*ICAM1*), as well as the IL-2 receptor subunits *IL2RA* and *IL2RB*, which were selected due to the continuous IL-2 supplementation during NK-cell cultivation (Figure 6).

Analysis of the variant distribution within the genomic regions of the selected genes revealed that the highest number of variants was detected in the IL-2 receptor subunits *IL2RA* and *IL2RB*. The majority of these variants represented heterozygous genotypes with one reference and one alternate allele, followed by homozygous alternate genotypes, whereas heterozygous genotypes with two different alternate alleles were rare. Upon normalization for gene length, a variant density of approximately two variants per kilobase (ranging from 1.6 to 2.4 variants/kb) was observed across all donors for *IL2RA* and *IL2RB*, except for donor 5, who displayed a slightly lower density in *IL2RB* (1.4 variants/kb).

*FCGR3A*, which encodes CD16a, exhibited notable donor-specific variation. Variant counts in this region revealed that donor 1 harbored the highest number of variants (21), followed by donor 5 (14), while donor 2 exhibited the fewest (7). Across all donors, most identified variants corresponded to heterozygous genotypes with one reference and one alternate allele, with the exception of donor 6, who additionally displayed two homozygous alternate variants. Upon normalization for gene length, donor 1 exhibited the highest variant density of 2.5 variants per kilobase, while the remaining donors ranged from 0.8 to 1.7 variants per kilobase. Across all analyzed genomic regions, the highest variant density observed for a single donor was detected in donor 1 within the genomic region of *NCR1*, reaching 12.4 variants per kilobase. In the remaining donors, variant densities ranged from 6.0 to 6.8 per kilobase. In donors 1 and 2, most variants corresponded to heterozygous genotypes with one reference and one alternate allele, whereas donors 3, 4, and 5 predominantly exhibited homozygous alternate genotypes. Additionally, donors 3, 4, and 5 each harbored one heterozygous variant involving two different alternate alleles. The genomic region of *KLRK1*, associated with NKG2D expression, displayed the highest overall proportion of homozygous alterations, particularly in donors 1, 2, and 3. Donor 4 presented a mixed pattern, comprising 25 heterozygous variants with one reference and one alternate allele and 21 homozygous alternate variants. In contrast, donor 5 exhibited 29 heterozygous variants with one reference and one alternate allele, along with a single homozygous alternate variant and one heterozygous variant involving two different alternate alleles. Such variants were also observed in donors 1 and 2 (one each) and in donor 4 (four variants), while donor 3 did not harbor any. Normalization of the number of variants within the genomic region of *KLRK1* to the gene length revealed densities ranging from 2.5 to 2.8 variants per kilobase across donors, except for donor 5, who exhibited a lower density of 1.8 variants per kilobase.

Within the genomic region of *ICAM1*, variant densities ranged only from 0.8 to 1.3 variants per kilobase across donors. Heterozygous genotypes with one reference and one alternate allele were most common in terms of absolute variant count, except for donor 2, exhibiting nine heterozygous variants compared to eleven homozygous alternate variants. Among the other donors, only one or two homozygous alternate variants were detected, and donor 3 additionally displayed one heterozygous variant involving two different alternate alleles.

To further investigate potential functional relevance, a refined subset of high-priority SNPs was selected, including only predicted missense variants classified to have at least moderate functional impact (Figure 6C, Table 3). Within the CD16a receptor gene (*FCGR3A*), three high-priority SNPs were identified. The SNP rs115866423 was exclusively detected in donor 4, carrying the variant in a heterozygous state. Donors 1 and 3 carried rs396991 in a heterozygous state with one reference and one alternate allele, whereas donor 5 was homozygous for the alternate allele at this position. In addition, donor 5 also carried rs10127939 in a heterozygous state. In contrast, donor 2 carried reference alleles at all three loci, with no indication of the corresponding variants. For the other targeted receptor genes, only one high-priority SNP per gene was identified, except for *IL2RA*, for which no such variant was detected. Within the genomic region of *KLRK1*, encoding NKG2D, the identified SNP rs2255336 was present in a homozygous state with two alternate alleles in all donors except for donor 5, who carried one reference and one alternate allele. A similar distribution was observed for rs2278428 within the genomic region of *NCR1*, which encodes NKp46. Donors 1, 3, and 5 were homozygous for the alternate allele, donor 4 carried one reference and one alternate allele, and the variant was absent in donor 2. The high-priority SNP detected within the genomic region of *ICAM1* (rs5498) was found in a heterozygous state exclusively in donors 3 and 5.

## 4. Discussion

For NK-cell research and its successful therapeutic application, a critical prerequisite is the development of robust ex vivo expansion protocols that yield functional NK cells in sufficient numbers [35]. While many studies focus on the optimization of cytokine cocktails or the comparison of activating compounds, fundamental cultivation parameters such as the initial seeding density have received comparatively little attention [35,36,37]. Despite their potential impact on proliferation dynamics and phenotypic stability, relevant literature addressing these basic aspects remains scarce [38,39]. Furthermore, intrinsic donor variability can introduce considerable heterogeneity into experimental outcomes, compromising both reproducibility and translational applicability [38,40,41]. Understanding these interindividual differences is, therefore, essential for both basic research and clinical implementation, as they may influence both the functional output and the therapeutic potential of expanded NK cells [40,42].

This study aimed to investigate how initial seeding density and donor-related variability influence the proliferative capacity and surface receptor expression of in vitro-expanded primary human NK cells. To address this, NK cells from healthy donors were cultivated under defined conditions using the G-Rex^®^ system, and phenotypic changes were monitored over time by flow cytometry. Subsequently, a targeted SNP screen was conducted to explore whether genetic variations in receptor genes might account for observed inter-donor differences. These data were used to assess the relevance of seeding density as a cultivation parameter, define optimal conditions for NK-cell expansion in the G-Rex^®^ 24-well plate format, and better understand how donor-intrinsic factors may influence expansion outcomes and phenotypic profiles.

To evaluate the influence of seeding density on NK-cell proliferation, the following four initial input concentrations were tested: 0.5 × 10^6^, 1.0 × 10^6^, 2.0 × 10^6^, and 2.5 × 10^6^ NK cells/cm^2^. These densities were defined relative to the surface area of the G-Rex^®^ 24-well plate used in this study, which facilitates efficient gas exchange and nutrient availability through its gas-permeable membrane [6].

All cultures experienced a decline in cell counts during the early expansion phase. Although similar patterns have been observed with both mouse and human NK cells and different stimulation methods, the underlying mechanisms remain unclear, with the suggested explanations being autolysis, shift in NK-cell subsets, and activation-induced cell death, as well as adaptation stress [10,43,44,45]. Since the early decline in cell populations observed here is mainly apparent for viable lymphocytes and matches the time frame in which the highest increase in NK-cell purity was observed, most of this observation seems to be a result of the selective culture conditions and the associated loss of contaminating cell types. However, since the observed NK-cell expansion also experienced a drop below 1 in the lowest seeding density, missing cell–cell contacts likely play a role in this effect.

Cultures initiated with 2.0 × 10^6^ or 2.5 × 10^6^ NK cells/cm^2^ yielded significantly greater cell densities than those seeded at 0.5 × 10^6^ or 1.0 × 10^6^ cells/cm^2^, while no statistically significant difference was observed between the two highest seeding densities. This trend may be explained by enhanced cell-to-cell contact at higher seeding densities, which has been shown to promote IL-2 receptor expression and NK-cell proliferation during the early expansion phase [46].

This pattern was also reflected when normalizing to the initial input by calculating expansion. While the differences between seeding densities were less pronounced in this metric, a trend toward increased expansion at higher inputs remained evident, with peak values at 2.0 × 10^6^ NK cells/cm^2^ seeding density. Although this increase did not reach statistical significance, it suggests a potential advantage in total expansion capacity at higher inputs. Interestingly, a similar pattern was reported by Lapteva et al., who observed that while higher seeding densities led to greater absolute NK cell yields, the most efficient expansion relative to input cell number was achieved at 2.0 × 10^6^ NK cells per cm^2^. While both studies based their calculations on CD56^+^CD3^−^ NK cell numbers determined by flow cytometry, our cultures were initiated with pre-enriched NK-cell fractions, whereas Lapteva et al. used whole PBMCs as input material. The presence of accessory immune cells in the latter may have influenced cytokine availability, activation status, and overall expansion dynamics, thereby limiting direct comparability [47].

Notably, the observation that cultures seeded at 2.0 × 10^6^ and 2.5 × 10^6^ NK cells/cm^2^ reached similarly high peak densities and began to decline at comparable time points suggests that increasing seeding densities does not necessarily enhance expansion rate. However, the fact that cultures with higher seeding densities reached greater peak cell concentrations before declining from a similar time point suggests that the system itself permits higher NK-cell densities than those observed in this study.

In addition to its effect on proliferation, seeding density also modulated the expression of several key surface receptors. Overall, higher initial densities were associated with enhanced expression of the activating receptors CD16a, NKp46, and NKG2D, both in terms of the percentage of positive NK cells and MFI. This trend was particularly evident from day 8 onward, suggesting that higher cell densities promote or sustain receptor expression during the expansion phase.

In the case of CD16a, a rapid decline in expression was observed across all conditions immediately after seeding, likely reflecting the known IL-2-mediated downregulation of this receptor [48]. However, recovery of CD16a expression was more pronounced at higher densities, with the two highest seeding conditions consistently maintaining higher CD16a levels throughout the cultivation period. Similarly, NKp46 and NKG2D expression increased more steadily and reached higher levels in cultures initiated at 2.0 × 10^6^ and 2.5 × 10^6^ NK cells/cm^2^, supporting the notion that cell–cell interactions and local cytokine accumulation contribute to the maintenance or re-induction of activating receptor expression.

In contrast, ICAM-1 exhibited an inverse pattern. Although the percentage of ICAM-1^+^ NK cells was relatively comparable across seeding densities, the MFI was consistently highest at the lowest input (0.5 × 10^6^ cells/cm^2^). This suggests that at reduced cell densities, ICAM-1 may be upregulated to compensate for limited intercellular contact, in line with its known role in facilitating immune synapse formation and adhesion under conditions of sparse cell distribution [49]. While ICAM-1 increases the efficiency of target cell binding, it is not the only adhesion molecule involved in NK-cell interactions. Other molecules such as 2B4 can also mediate cell–cell contact and signaling [46]. Furthermore, NK-cell activation and cytotoxicity are governed by a balance of activating and inhibitory signals [2]. While reduced ICAM-1 expression was observed at higher seeding densities, the concurrent upregulation of key activating receptors such as CD16a, NKp46, and NKG2D may outweigh this disadvantage, given their more direct roles in NK-cell activation and cytotoxic function [13,14,15]. Additionally, homotypic interactions via molecules such as 2B4/CD48 support NK-cell survival and activation independently of ICAM-1, further reducing the reliance on ICAM-1-mediated adhesion [46].

Taken together, these findings highlight that initial seeding density plays a critical role not only in determining the overall expansion capacity of NK cells but also in shaping their phenotypic profile. Higher input densities were associated with greater total yields and more sustained expression of key activating receptors such as CD16a, NKp46, and NKG2D. In the context of translational NK-cell manufacturing, a seeding density of 2.0 × 10^6^ NK cells/cm^2^ may represent an optimal compromise between expansion performance and cell availability in the G-Rex^®^ system, particularly when starting material is limited. These results illustrate that seeding density is a key parameter influencing both the quantitative and functional quality of ex vivo expanded NK-cell products.

While optimizing seeding density can improve NK-cell expansion and function, it is crucial to acknowledge the significant impact of donor-to-donor variability, even under controlled culture conditions [40,42]. Understanding these donor-specific factors is essential for developing robust and reliable NK-cell expansion protocols for clinical applications. Accordingly, the following section characterizes inter-donor differences in terms of proliferation, receptor expression, and relevant receptor gene polymorphisms.

Donors 2 and 3 exhibited superior proliferation, with donor 3 additionally showing the highest NK-cell purity across the entire culture period. Although donor 2 started with lower purity, it reached comparable levels from day 15 onward. Donor 3 exhibited high initial and sustained expression of CD16a and NKp46, while NKG2D expression remained average. ICAM-1 levels were initially elevated but declined to intermediate values after day 8. Donor 2 exhibited the lowest ICAM-1 and NKG2D expression at baseline, but both increased during culture. CD16a expression remained comparatively low throughout. It is well established that CD16a expression varies considerably among healthy individuals [50]. While the underlying mechanisms are not fully understood, epigenetic regulation, prior immune activation, and potentially genetic polymorphisms may contribute to inter-individual variation in NK-cell responsiveness [51,52]. Genetic polymorphisms within the *FCGR3A* gene, which encodes CD16a, is known to influence both receptor expression and function [53]. While specific SNPs identified in this study are discussed in more detail below, it is important to note that CD16a expression levels may be clinically relevant, especially in therapies relying on ADCC. Given CD16a’s role in mediating ADCC, selecting donors with high CD16a expression or favorable *FCGR3A* genotypes could enhance the efficacy of NK-cell-based immunotherapies [53,54]. NKp46 expression in donor 2 was unremarkable, showing no noteworthy deviation from the other donors.

In contrast to donors 2 and 3, donor 5 exhibited markedly reduced proliferative capacity and showed, despite having the highest initial NK-cell purity, the lowest purity from day 12 onward. Donor 5 also stood out due to a pronounced reduction in NKp46 expression. Although initial levels were comparable to those of other donors, the proportion of NKp46^+^ cells declined to only 16% by day 25, whereas all other donors reached values between 83% and 95%. ICAM-1 expression was also lowest in donor 5, with only 53% ICAM-1^+^ cells on day 25, compared to 91–97% in the other donors. CD16a levels were within the cohort range, while NKG2D expression was elevated, especially at day 8.

To determine whether these differences could be attributed to underlying genetic variation, a targeted SNP analysis was subsequently performed. A notable finding in the genetic profile of donor 5 was the presence of the high-priority SNP rs2255336 in the *KLRK1* gene, which encodes the activating receptor NKG2D. This SNP results in a missense variant at amino acid position 72, substituting threonine (Thr) with alanine (Ala) (p.Thr72Ala) in the protein product (NP_001186734.1) [55]. This residue marks the terminal position of the transmembrane region (residues 52–72), placing the substitution exactly at the boundary between the membrane anchor and the extracellular domain, a location known to influence receptor orientation and signal initiation [56,57]. While all other donors in this study were homozygous for the common alternate allele (Ala/Ala, encoded by the C allele), donor 5 was heterozygous, carrying one reference and one alternate allele, resulting in a threonine at this critical boundary position. Population data indicate that this T allele is found in only ~21% of individuals, whereas the C allele (coding for alanine) has a frequency of ~79%, highlighting donor 5 as genetically distinct both within the study cohort and relative to global distributions [55]. This variant has been implicated in disease-associated immune modulation across multiple independent studies. In patients with chronic hepatitis B, the Thr72 variant (T allele) was associated with increased disease susceptibility, potentially due to altered NKG2D-mediated cytotoxicity and immune recognition [58]. Similarly, in systemic lupus erythematosus, carriers of the T allele displayed modified NK and CD8^+^ T-cell responsiveness, suggesting that the variant affects downstream immune activation [59]. A third study in patients with rheumatoid arthritis reported that the same allele influenced the response to TNF-α inhibitors, supporting the notion of altered receptor signaling dynamics [60].

In our study, donor 5 exhibited elevated surface expression of NKG2D, particularly during early cultivation. At the same time, donor 5 demonstrated reduced proliferative capacity raising the possibility of impaired signal transduction despite high receptor abundance. One explanation might be a compensatory upregulation of receptor expression in response to impaired ligand interaction or suboptimal receptor anchoring, potentially resulting from altered conformation at the transmembrane boundary. While direct evidence for such compensatory upregulation of NKG2D in the context of genetic variants is lacking, the observed expression profile in donor 5 may reflect a cellular attempt to restore signaling capacity under conditions of reduced receptor functionality.

Notably, donor 4, despite a similar number of heterozygous (Ref/Alt) variants in the *KLRK1* region, did not carry the T allele and showed neither altered NKG2D expression nor reduced expansion, reinforcing the functional relevance of this variant. While donor 4 exhibited a more heterogeneous genotype profile, including multiple homozygous alternate variants and heterozygous positions involving two distinct alternate alleles, these did not overlap with the reference-allele-specific positions found in donor 5. Donor 5 uniquely carried multiple reference alleles at distinct loci within *KLRK1*, suggesting a haplotypic pattern not shared with any other donor, which may enhance the functional relevance of rs2255336 (Table A1). Notably, these included variants, annotated as having low or modifier impact, which individually may not be functionally prioritized but could gain relevance through their combined presence. This interpretation is supported by findings in the *IL1B* promoter, where promoter activity depends not on individual SNPs alone but on their combined arrangement within specific haplotypes [61].

In addition to its distinct genotype pattern in *KLRK1*, donor 5 also harbored two rare variants within the *FCGR3A* gene, which encodes CD16a, a low-affinity Fcγ receptor for IgG antibodies [14]. Among these, rs396991 represents a well-studied missense SNP that causes a phenylalanine (Phe) to valine (Val) substitution at position 176 of the receptor protein (p.Phe176Val in current NCBI annotation; historically referred to as Val158Phe) [62]. Donor 5 was homozygous for the alternate allele (Val/Val), while donors 1 and 3 were heterozygous (Phe/Val), and donors 2 and 4 carried the homozygous reference genotype (Phe/Phe). This SNP has been functionally linked to altered CD16a (FcγRIIIa) affinity and NK-cell-mediated ADCC, with the valine variant conferring increased IgG1 binding and enhanced cytotoxicity, particularly in the context of monoclonal antibody therapies [63].

In addition, donor 5 uniquely carried a second rare missense SNP within *FCGR3A*, rs10127939, resulting in a leucine-to-histidine substitution at position 167 of CD16a (p.Leu167His) [64]. This variant was present in a heterozygous state (A/T) exclusively in donor 5, while all other donors were homozygous for the reference allele (A/A). The T allele, encoding histidine, is relatively rare in the general population, with a minor allele frequency of ~4.6% and homozygosity observed in only 0.03% of individuals [64].

The functional relevance of rs10127939 has been demonstrated in patients with primary immunodeficiencies. Grier et al. described the same substitution—referred to as L66H under an alternative numbering scheme—in individuals with impaired NK-cell cytotoxicity but preserved ADCC, suggesting that this variant may disrupt spontaneous NK-cell activation [65]. Previous studies reported a similar substitution in patients with NK-cell deficiency and recurrent infections [66,67].

Interestingly, despite carrying rs10127939 in heterozygous form and rs396991 in homozygous form, donor 5 showed CD16a expression levels within the upper range of the cohort. Donors 1 and 3, both heterozygous for rs396991, also exhibited elevated CD16a MFI compared to donors 2 and 4. Notably, donor 3 displayed the highest CD16a expression despite sharing the same rs396991 genotype with donor 1, who showed only moderate levels. This variation suggests that the presence of rs396991 alone is insufficient to predict CD16a expression outcomes and that additional genetic or regulatory factors are likely involved. Specifically, the differential expression between donors 1 and 3, who share the same rs396991 genotype, underscores the influence of other modifiers. A study by Pérez-Romero et al. reported that rs10127939 and rs396991 can influence CD16a surface recognition and NK-cell subset distribution, supporting the idea that SNP combinations may influence receptor expression in a context-dependent manner [68]. Furthermore, the finding that donor 5 exhibited high CD16a expression, despite carrying both rs10127939 and rs396991, suggests a more complex regulatory interplay beyond single-SNP effects. A functional ADCC assay would provide valuable insights into the relationship between CD16a surface expression, the *FCGR3A* genotype, and NK-cell effector function. Considering the observed donor-specific differences in CD16a expression and the presence of functionally relevant *FCGR3A* variants, this assay would be particularly informative to determine whether these phenotypic and genetic differences translate into altered functional responses.

Among the analyzed activating receptors, NKp46 expression in donor 5 displayed the most pronounced decline during culture. While the initial proportion of NKp46^+^ cells was within the range of the other donors, expression decreased progressively over time rather than increasing or stabilizing. By day 25, only 16% of NK cells in donor 5 remained NKp46^+^, whereas values in the remaining donors ranged from 83% to 95%. This represents the most substantial receptor reduction observed across all donors. This result was unexpected, as all other donors maintained stable or increasing NKp46 levels under identical IL-2 stimulation. IL-2 has been shown to support NKp46 expression in cultured NK cells from healthy individuals [69]. Reduced NKp46 expression has previously been described in disease contexts such as gastric cancer and non-small-cell lung cancer, where it often coincides with suppressed NK-cell activity and disease progression [70,71]. Unlike these cases, donor 5 initially exhibited average NKp46 levels but showed a progressive loss under cytokine-supported expansion. In healthy donors, NKp46 is generally maintained under IL-2 culture, and no consistent downregulation has been reported [69]. Thus, the underlying cause of this donor-specific pattern remains unclear.

Given the absence of a high-priority SNP in the *NCR1* gene unique to donor 5, genetic factors appear unlikely to explain the observed NKp46 loss. Alternative mechanisms such as altered NK-cell subset composition or regulatory processes at the transcriptional level may instead contribute. Previous studies have demonstrated that NK-cell receptor expression is dynamically regulated through transcriptional and epigenetic mechanisms, particularly in the context of cytokine stimulation [72]. Epigenetic modifications in the *NCR1* promoter region, particularly DNA methylation, have been identified as key modulators of NKp46 expression. For example, hypomethylated CpG sites within the *NCR1* locus correlated with NK-cell abundance and effector functions, suggesting that cell-intrinsic epigenetic states can modulate receptor profiles [72]. These findings support the hypothesis that the pronounced NKp46 downregulation observed in donor 5 could stem from context-dependent, epigenetically mediated transcriptional repression rather than from static genetic variation.

Strikingly, this aberrant pattern was not limited to NKp46. Donor 5 also showed markedly reduced ICAM-1 expression during late cultivation. Although initial levels were moderate, expression continuously declined to only 53% ICAM-1^+^ cells by day 25, substantially lower than in all other donors (91–97%). This finding stands in contrast to results from the seeding density experiments, where lower cell densities were consistently associated with elevated ICAM-1 surface expression. Given that donor 5 proliferated poorly and therefore maintained a lower cell density throughout the culture period, one would have expected an increase in ICAM-1 levels. However, the opposite was observed, suggesting an uncoupling of this density-dependent regulatory mechanism in donor 5. Together with the NKp46 pattern, the sustained ICAM-1 reduction may indicate a broader dysfunction in receptor regulation, potentially involving epigenetic or transcriptional dysregulation affecting NK-cell effector function [51,52,72].

While donor 5 stood out due to a combination of impaired proliferation and aberrant receptor expression, potentially linked to its unique genetic background, donors 2 and 3 presented a contrasting phenotype. Both exhibited consistently high proliferative capacity, clearly outperforming the other donors. However, despite this shared advantage, it remained unclear whether similar genetic factors contributed to their enhanced expansion, warranting a closer examination of their genotypic profiles. Phenotypic profiles, including activating receptor expression, were assessed first to determine whether observable differences accompanied the enhanced expansion, before addressing potential genetic factors.

Donor 3 was characterized by particularly elevated expression of activating receptors. CD16a surface density (MFI) was the highest among all donors and already markedly elevated upon isolation. NKp46 followed a similar trajectory, starting at a high level and remaining stably elevated throughout cultivation. ICAM-1 expression in donor 3 was initially above average and reached the second-highest value by day 8, declining thereafter. NKG2D expression was comparatively average.

Genetically, donor 3 harbored five high-priority SNPs with alternate alleles, as follows: rs228942 (*IL2RB*), rs396991 (*FCGR3A*), and rs5498 (*ICAM1*) in heterozygous form, as well as rs2278428 (*NCR1*) and rs2255336 (*KLRK1*) in homozygous form. Given the central role of IL-2 in NK-cell proliferation, one might have expected variation in *IL2RA* or *IL2RB*—either enhancing signaling in donors 2 and 3 or impairing it in donors like donor 5 [73]. Although a high-priority SNP was identified in *IL2RB* (rs228942), it was shared by donor 1 and donor 3 in heterozygous form, while donor 2 carried the common reference allele, making a shared genetic basis for enhanced proliferation unlikely. Moreover, none of the high-priority SNPs in donor 3 were unique to this individual, and no consistent genotypic pattern emerged that could explain the proliferation advantage.

Donor 2 displayed a slightly different phenotype. Although its initial NK-cell purity was lower, a rapid increase during cultivation placed donor 2 above average for NK-cell enrichment. Receptor expression, however, was less pronounced. CD16a and NKp46 remained within the average range. ICAM-1 expression was among the lowest throughout the culture period, while NKG2D, initially expressed at the lowest level, rose steeply to surpass most donors by day 8.

Genetically, donor 2 carried no unique high-priority SNPs. Like donor 3 and most other individuals, donor 2 was homozygous for the alternate allele of rs2255336 (*KLRK1*), which was previously discussed in detail due to its heterozygous state in donor 5. Since this genotype was broadly shared and represents the globally more common variant, it is unlikely to account for the superior proliferation observed in donors 2 and 3 [55].

Taken together, the proliferation advantage observed in donors 2 and 3 may reflect shared underlying mechanisms. However, neither a common high-priority SNP nor a consistent receptor profile emerged that could conclusively explain their phenotype. This underscores the challenge of linking NK-cell behavior to single genetic variants and highlights the need for integrative analyses considering both genetic and non-genetic influences on proliferation.

Beyond static variation, one plausible explanation may be the expansion of adaptive NK-cell subsets in response to cytomegalovirus (CMV). CMV-seropositive individuals frequently develop CD57^+^NKG2C^+^ NK-cell subsets with epigenetic remodeling and enhanced cytokine responsiveness [52,74]. These adaptive NK cells proliferate robustly in response to IL-2, matching the expansion profiles seen in donors 2 and 3. Although CMV serostatus was not determined in this study, the possibility that these donors harbored adaptive NK-cell subsets cannot be excluded and could partially explain their heightened proliferation under IL-2 stimulation.

A second explanation may involve donor-specific differences in epigenetic priming. IL-2 stimulation induces histone acetylation at effector gene enhancers such as interferon gamma, and broader epigenetic variability, potentially shaped by factors like age, environmental exposure, or infection history, may influence NK-cell responsiveness [75].

Thus, these considerations suggest that the enhanced expansion seen in donors 2 and 3 may stem from intrinsic functional properties of their NK-cell populations, potentially shaped by previous immune stimulation or epigenetic programming, rather than from static genetic differences alone [51,52,72,74].

Taken together, this study highlights the relevance of both experimental parameters and donor-intrinsic factors in shaping NK-cell proliferation and phenotype during in vitro expansion. A seeding density of 2.0 × 10^6^ cells/cm^2^ was identified as a practical compromise between input availability and expansion efficiency, supporting high cell yields and favorable receptor expression profiles. This result was obtained using a G-Rex^®^ 24-well plate, which offers enhanced gas exchange and nutrient availability [6]. While the findings are specific to this format, G-Rex^®^ technology is broadly applied in translational NK-cell protocols due to its scalability and compatibility with clinical manufacturing [5,7,8].

Despite standardized culture conditions, pronounced inter-donor variability was observed. Donor 5, in particular, exhibited impaired expansion and aberrant receptor expression, potentially reflecting a combination of genetic and regulatory influences. In contrast, donors 2 and 3 demonstrated robust expansion without identifiable genetic correlates, suggesting that non-genetic mechanisms such as adaptive differentiation or epigenetic priming may underlie their enhanced responsiveness [74,75].

The genetic analysis focused on a predefined panel of NK-cell receptor genes, enabling detailed correlations between specific variants and receptor expression phenotypes. Although no definitive genotype–phenotype relationships could be established due to the modest cohort size, selected high-priority SNPs, such as rs2255336 (*KLRK1*), rs396991, and rs10127939 (*FCGR3A*), revealed donor-specific patterns with potential biological relevance [59,60,63,65,66,67,68]. Furthermore, variants annotated with low or modifier impact may reflect underlying haplotype structures, particularly in donor 5, highlighting the value of integrating broader genomic and regulatory information into future analyses. From a translational perspective, these findings underscore the importance of optimizing baseline parameters, such as seeding density to improve the consistency and quality of NK-cell products. At the same time, they highlight the potential of integrated donor characterization, encompassing phenotypic, genetic, and potentially epigenetic profiling, for future efforts in personalized NK-cell therapy and transplantation. Incorporating SNP analysis into donor selection workflows may, in the long term, help predict cell product quality, guide therapeutic strategies, and reduce inter-individual variability in clinical outcomes.

Future studies should expand on these findings by including larger donor cohorts and functional assays such as cytotoxicity testing, cytokine release, and, in particular, ADCC, to evaluate how phenotypic and genotypic differences translate into effector function. Broadening the genetic analysis beyond receptor gene variants, ideally through whole genome or exome sequencing, may reveal additional regulatory or metabolic pathways involved in NK-cell proliferation [76]. In addition, incorporating analyses of epigenetic regulation, CMV serostatus, and NK-cell differentiation states would further deepen our understanding of donor-specific NK-cell behavior and support the refinement of expansion protocols for clinical use.

## 5. Conclusions

This study demonstrates that initial seeding density and intrinsic biological variation critically shape the outcome of in vitro NK-cell expansion. Within the G-Rex^®^ 24-well format, a seeding density of 2.0 × 10^6^ cells/cm^2^ provided optimal conditions for NK-cell proliferation and the maintenance of activating receptor expression, including CD16a, NKp46, and NKG2D. Despite standardized culture conditions, pronounced differences in expansion dynamics and receptor profiles were observed between individual NK-cell donors. While some of these differences coincided with distinct genetic constellations, others occurred in the absence of identifiable genetic correlations. This suggests that genetic background alone is insufficient to explain the full extent of variability and points to additional influences such as epigenetic priming, transcriptional regulation, or prior immune stimulation. These findings highlight the importance of integrating phenotypic and genetic analyses to better understand the regulatory mechanisms underlying NK-cell behavior and to advance the development of standardized yet adaptable manufacturing strategies. Future studies incorporating broader genomic, transcriptional, and functional profiling may further elucidate the determinants of NK-cell quality and support the development of consistent and personalized NK-cell-based immunotherapies.

## Figures and Tables

**Table 1:** NK-cell donor characteristics. The table summarizes sex, year of birth, and the number of isolated NK cells per mL of buffy coat for each donor. All blood donations were performed on 2 August 2023.

Donor	Sex	Year of Birth	NK Cells per mL Buffy Coat
1	male	1968	1.3 × 10^6^
2	female	1990	2.8 × 10^6^
3	female	1972	1.1 × 10^6^
4	female	1976	0.7 × 10^6^
5	male	1977	1.2 × 10^6^

**Table 2:** Overview of antibodies and dyes used in the 8-color flow cytometry panel for the phenotypic characterization of NK cells during long-term culture. The table includes marker names, clones with fluorochromes, working dilutions, and functional classification within the panel. All antibodies were obtained from Miltenyi Biotec (Bergisch Gladbach, Germany); the viability dye (4’,6-diamidin-2-phenylindol, DAPI) was purchased from Sigma-Aldrich (Steinheim, Germany).

Marker	Monoclonal Antibody/Fluorochrome	Dilution	Function in NK Panel
CD45	CD45 antibody, anti-human VioGreen, REA747	1:50	Identification of hematopoietic cells
CD3	CD3 antibody, anti-human PE, REA613	1:50	Exclusion of T cells and NKT cells
CD56	CD56 antibody, anti-human APC-Vio770, REA196	1:50	Identification of NK cells
CD16a	CD16 antibody, anti-human PE-Vio615, REA423	1:50	Fc receptor mediating ADCC
NKp46 (CD335)	CD335 (NKp46) antibody, anti-human Vio Bright, REA808	1:25	Natural cytotoxicity receptor
NKG2D (CD314)	CD314 (NKG2D) antibody, anti-human PE-Vio 770, REA797	1:25	Stress-induced ligand receptor
ICAM-1 (CD54)	CD54 (ICAM-1) antibody, anti-human PE-APC, REA266	1:50	Adhesion molecule
Viability	DAPI diluted in PBS containing 2 mM EDTA and 2% FCS	1:20,000	Exclusion of dead cells

**Table 3:** Overview of selected high-priority SNPs identified in sequenced donor samples. Variants were classified as high-priority based on a predicted missense effect and a moderate predicted functional impact. The table lists the affected gene, dbSNP identifier (rsID), reference and alternate alleles (Ref/Alt), and the genotype for each donor [34].

Gen	rsID	Ref/Alt	Donor				
			1	2	3	4	5
*IL2RB*	rs228942	G/T	0/1	0/0	0/1	0/0	0/0
*FCGR3A*	rs115866423	T/A	0/0	0/0	0/0	0/1	0/0
*FCGR3A*	rs396991	A/C	0/1	0/0	0/1	0/0	1/1
*FCGR3A*	rs10127939	A/T	0/0	0/0	0/0	0/0	0/1
*NCR1*	rs2278428	C/A	1/1	0/0	1/1	0/1	1/1
*KLRK1*	rs2255336	T/C	1/1	1/1	1/1	1/1	0/1
*ICAM1*	rs5498	A/G	0/0	0/0	0/1	0/0	0/1

## Data Availability

Raw data are available upon request from the corresponding author.

## Abbreviations

The following abbreviations are used in this manuscript:

ADCC	Antibody-Dependent Cellular Cytotoxicity
Ala	Alanine
Alt	Alternate Allele
CMV	Cytomegalovirus
DAPI	4′,6-diamidino-2-phenylindol
DMSO	Dimethyl Sulfoxide
EDTA	Ethylenediaminetetraacetic Acid
FCS	Fetal Calf Serum
FcγRIIIa	Fc Gamma Receptor IIIa (CD16a)
gDNA	Genomic DNA
ICAM-1	Intercellular Adhesion Molecule 1
IL-2	Interleukin-2
MFI	Mean Fluorescence Intensity
NCR	Natural Cytotoxicity Receptor
NK	Natural Killer
NKG2D	Natural Killer Group 2, Member D
ONT	Oxford Nanopore Technologies
PBS	Phosphate-Buffered Saline
PBSE	Phosphate-Buffered Saline + EDTA
Phe	Phenylalanine
Ref	Reference Allele
SNP	Single-Nucleotide Polymorphism
Thr	Threonine
Val	Valine

## Appendix A

**Table A1:** Global allele and genotype frequencies of selected SNPs within the genomic region of *KLRK1*. The table includes variants for which donor 5 carried the heterozygous genotype (Ref/Alt), while donors 1–4 were homozygous for the alternate allele (Alt/Alt). For each SNP, the dbSNP identifier (rsID), reference and alternate alleles, and their global allele frequencies are listed, along with the genotype distribution based on data from the NCBI dbSNP database [34].

rsID	Ref/Alt	Global Frequency				
		Ref	Alt	Ref HMOZ ^1^	Alt HMOZ ^1^	HTRZ ^2^
rs2255364	C/T	0.23449	0.76551	0.06508	0.596095	0.338824
rs2255353	T/G	0.22793	0.77207	0.062474	0.606614	0.330912
rs2255336 ^3^	T/C	0.214396	0.785604	0.050658	0.621866	0.327475
rs2617151	A/G	0.194963	0.805037	0.038508	0.648582	0.31291
rs2733848	G/T	0.23504	0.76496	0.065846	0.595766	0.338388
rs2617152	T/C	0.23567	0.76433	0.068484	0.597145	0.334371
rs2733847	T/C	0.2811	0.7189	0.146492	0.584365	0.269143
rs2417734	T/A	0.2626	0.7374	0.127589	0.602316	0.270096
rs1382265	T/C	0.23487	0.76513	0.065697	0.595952	0.33835
rs2733853	A/G	0.23441	0.76559	0.065025	0.596215	0.338761
rs1478310	C/T	0.23545	0.76455	0.065615	0.59471	0.339674
rs1600127	T/C	0.23535	0.76465	0.06824	0.597532	0.334227
rs2617156	A/G	0.23513	0.76487	0.064972	0.594713	0.340315
rs2617157	C/T	0.2815	0.7185	0.146587	0.583601	0.269812
rs2927561	A/G	0.23416	0.76584	0.064667	0.59634	0.338993
rs2126889	C/A	0.2811	0.7189	0.146459	0.584231	0.26931
rs2733845	G/C	0.19434	0.80566	0.038751	0.650079	0.31117
rs1478309	T/G	0.21156	0.78844	0.058309	0.635185	0.306506
rs2045876	C/T	0.19590	0.80410	0.039333	0.647525	0.313142

^1^ Homozygous; ^2^ Heterozygous; ^3^ High-priority SNP.
